# Comparative analysis of the degree of patient satisfaction after breast-conserving surgery with or without oncoplastic surgery: systematic review and meta-analysis

**DOI:** 10.3389/fsurg.2024.1396432

**Published:** 2024-07-16

**Authors:** Fabiana Christina Araújo Pereira Lisboa, Lucimara Priscila Campos Veras Giorgi, Ana Claudia Morais Godoy Figueiredo, Régis Resende Paulinelli, João Batista de Sousa

**Affiliations:** ^1^ School of Medicine, University of Brasília (UnB), Brasília, Brazil; ^2^ Teaching and Research Foundation in Health Sciences (FEPECS), Brasília, Brazil; ^3^ School of Medicine, Federal University of Goiás, Goiânia, Brazil

**Keywords:** breast cancer, mammoplasty, conservative surgery, patient satisfaction, oncoplastic surgery

## Abstract

**Introduction:**

Conservative surgery is the gold standard for the treatment of single and small tumors and, combined with the concept of oncoplastic tumors, brings good aesthetic results while maintaining cancer safety. The objective was to comparatively analyze the degree of satisfaction of patients undergoing breast conserving surgery (BCS), with and without oncoplastic surgery (OPS) using level II OPS techniques.

**Methods:**

Review with a search in the databases MEDLINE (by PubMed), EMBASE, Clinical Trials, Scopus, Web of Science, BVS and Oppen gray. The meta-analysis of random effects was performed using the Der Simonian-Laird method considering the odds ratio (OR) with a 95% confidence interval (95% CI).

**Results:**

There was no statistically significant difference in the aesthetic outcome between women who underwent OPS and BCS (OR 0.90; 95% CI 0.62–1.30). The staging (OR 1.93; 95% CI 0.97–3.84; *I*^2^ = 15.83%); tumor location [central (OR 1.28; 95% CI 0.06–27.49; *I*^2^ = 17.63%); lower (OR 0.75; 95% CI 0.21–2.65; *I*^2^ = 2.21%); superior (OR 0.67; 95% CI 0.26–1.74; *I*^2^ = 0.00%] and tumor size (OR 8.73; 95% CI −11.82–29.28; *I*^2^ = 93.18%) showed no association with the type of BCS performed, with or without OPS. The degree of satisfaction remains even in cases of extreme oncoplasty.

**Conclusion:**

The level of patient satisfaction in relation to BCS was similar to that of the group undergoing OPS, highlighting that OPS allows the patient's satisfaction rate to be maintained even in the case of large or multicentric tumors.

## Introduction

The surgical treatment of breast cancer has always carried the stigma of sequelae and major deformities with a negative impact on aesthetics and quality of life. The BCS brought a change in this paradigm with a new perspective in breast surgery becoming the surgery of choice in the treatment of breast cancer (1, 2). However, up to 30% of people undergoing quadrantectomy require other corrective surgeries due to unsatisfactory aesthetic results (3), and these late corrections can be avoided with the use of oncoplastic techniques (4), which allow the removal of large breast volumes and at the same time a better aesthetic result. OPS was created in the 1980s to combine cosmetic surgery techniques with oncologic surgery (3) and made it possible to expand the indications for conservative breast treatment for women whose tumor/breast size ratio may have been previously prohibitive (5), especially in individuals with large tumors, which may prevent mastectomy in some cases, multifocal/multicentric tumors, tumors in areas of high risk of deformity (lower pole, internal quadrants of the breast) (1), cancer safety (4, 6, 7) and the same overall survival (3).

The indication for OPS has increased in the proportion in which surgeons gain experience with different oncoplastic techniques and better aesthetic results when offering contralateral symmetrization (6) and is related to tumor location, breast volume, tumor size, need for breast reduction and ptosis correction (3). Mastopexy, performed in the resection of the tumor, allows the repositioning of the areola-papillary complex and offers a chance of simultaneous breast augmentation with prosthesis, possibly offering a more beneficial subjective result (8). In addition to the aesthetic benefit, there is the possibility of a broader resection (5), larger than possible in conventional surgery, with a large amount of resected tissue without aesthetic damage (6), in addition to a greater possibility of free margins. Other aspects that should be considered are the lower re-excision rate and a risk of complications that seem similar to the classic conservative treatment (8). The reduction of breast size using oncoplastic techniques can bring benefits by minimizing the undesirable effects of radiation (1), such as chronic pain, radiation toxicity to the skin, vasculitis and fibrosis of the breast parenchyma, which are more intense in larger breast volumes. In addition, it does not seem to negatively affect the duration of treatment and the occurrence of recurrence (5).

BCS is the gold standard for the treatment of early breast and has the clear advantage of shorter surgical time, lower cost and absence of treatment on the opposite side; however, clinical experience shows that some people appreciate the combined removal of the tumor together with a technique that allows a better aesthetic result that preserves the anterior shape of the breast (8–11). It is possible to opt for a conservative intervention in single and small breast tumors, with a good tumor/breast ratio and the possibility of performing BCS without leaving large surgical defects. Oncoplastic surgery may make it possible to perform conservative treatment in large, locally advanced or multicenter tumors in cases where mastectomy would be the most common treatment (12, 13). Several studies have shown comparable oncological results in breast cancer patients treated with conservative surgery and mastectomy, but few studies have compared conventional conservative surgery with oncoplastic surgery. The aesthetic result is one of the main objectives of oncoplastic surgery; therefore, its measurement is fundamental for understanding its impact on the individual's life (6). There are many measurement tools (software, questionnaires), with analyses at different scales from subjective evaluations to objective and valid methods (6, 14) requiring practical, objective and reproducible standardization (15). The comparative data between the aesthetic results in conservative surgery and oncoplastic surgery are limited (6, 16) in addition to being contradictory (14, 17). Previous research shows that questionnaires of aesthetic results reported by the patient provide more favorable data regarding the aesthetic result than objective measurements (14).

 The objective of the present study was to comparatively analyze the degree of satisfaction of patients undergoing breast-conserving surgery for breast cancer treatment, with level II OPS techniques and without oncoplastic surgery (conventional), in addition to conducting a meta-analysis to integrate the results of the independent studies conducted with different questionnaires to achieve a summary measure that could homogenize and categorize a complex comparative analysis to be evaluated with such diverse quantitative methods.

## Methods

### Registration and protocol

This systematic review was registered in the International Prospective Registry of Systematic Reviews (PROSPERO) under number CRD4202231310 and was conducted based on the Checklist for Meta-analyses of Observational Studies (MOOSE) (18) (Supplementary Appendix S1—MOOSE Checklist for Meta-analyses of Observational Studies) and Main Items for Reporting Systematic Reviews and Meta-analyses (PRISMA) (Supplementary Appendix S2—PRISMA checklist) (19). The Peer Review of Electronic Search Strategies (PRESS) checklist (Supplementary Appendix S3—PRESS Peer Review of Electronic Search Strategies) was applied, which is an instrument used for peer review of the search strategy (20). The Rayyan application was used, which is a free application that helps streamline the initial screening of abstracts and titles using a semi automation process, incorporating a high level of usability (21).

### Eligibility criteria

We included studies that reported patients with breast cancer who underwent conventional breast surgery and level II OPS techniques, evaluated the outcome of individual satisfaction with the aesthetic result in a comparative way and answered the evaluation questionnaires. Studies that did not perform comparative analysis and only evaluated the outcome satisfaction in isolation considering each type of surgery were excluded characterized as case series with fewer than 10 patients, with a short follow-up and in which it was not possible to safely analyze the outcome. For the meta-analysis, studies that did not report measures of association representing the outcome for the aesthetic satisfaction outcome were excluded.

### Sources of information and search strategy

Bibliographic searches were performed until June 2021. The following databases were used: MEDLINE (by PubMed), EMBASE, Clinical Trials, Scopus, Web of Science, BVS and Oppen gray (gray literature). The reference lists of the included records were manually searched for additional eligible articles. The authors were contacted for additional information needed.

The search strategy was developed using MeSH terms for PubMed and EMTREE terms for EMBASE, in addition to a combination of keywords for the other databases. The search strategy below was used in PubMed and subsequently adapted for each database (Supplementary Appendix S4—Search strategy for each database):

(((((((((((Mammaplasty [MeSH Terms]) OR (Mammaplasty [Title/Abstract])) OR (Mammaplasties [Title/Abstract])) OR (Mammoplasty [Title/Abstract])) OR (Mammoplasties [Title/Abstract])) OR (Breast Reconstruction [Title/Abstract])) OR (Breast Reconstructions [Title/Abstract])) OR (Reconstruction, Breast [Title/Abstract])) OR (Reconstructions, Breast [Title/Abstract]))) OR (Oncoplastic Surgery [Title/Abstract])) OR (Oncoplastic [Title/Abstract]).

No restrictions on date, publication status or type of study were applied. The selected articles were published in English.

### Study selection process

The selection of studies consisted first of the evaluation of the titles and abstracts after the removal of duplicate records. The evaluation of eligibility was performed by evaluating the full text, and articles that did not seem to meet the eligibility criteria were excluded. The data from the selected articles were extracted into a Microsoft Excel® 365 (2016) spreadsheet. The stages were performed independently by two authors (FCAPL and LPCV), and disagreements were resolved by consensus or with the intervention of a third reviewer, if necessary (ACMGF).

### Evaluation of study quality

The tools of the Joanna Briggs Institute were used to evaluate the methodological quality of the studies. The checklist for cohort studies (22) evaluated eleven questions related to the similarity between groups, measures of exposure and outcome, strategies to control for confounding factors, absence or presence of outcome at the beginning of follow-up, follow-up time and statistics. The greater the number of “yes” answers, the greater the probability of having good methodological quality.

### Quality of evidence and risk of bias

To grade the quality of the evidence and the strength of the recommendations, the GRADE evaluation was performed (23) which verifies five items that reduce the quality of the evidence by two points per item: risk of bias, inconsistency, indirect evidence, imprecision and publication bias. In parallel, three items increase the quality of evidence by up to two points: effect magnitude, dose‒response gradient and possible confusion adjustment. According to the GRADE classification, the evidence is considered of high quality when graded in at least four points, moderate quality in three points, low quality in two points and very low quality in one point (23).

We assessed the risk of bias of individual cohort studies included in this systematic review using the Newcastle‒Ottawa Quality Assessment Form for Cohort Studies tool (24). This instrument measures the representativeness of patient selection (representativeness of the cohort, selection of the unexposed cohort, determination of exposure and outcome of interest), comparability of the study groups, determination of outcomes, sufficient follow-up for the occurrence of the outcome and adequacy of the monitoring of cohorts. Each item has a score and allows classifying the studies as good, reasonable, or inadequate quality according to the degree of the domains.

### Data analysis

The primary outcome was the degree of patient satisfaction with the aesthetic outcome of breast-conserving surgery with or without oncoplastic surgery. The data were analyzed using STATA® version 17, serial number: 301709305247. To estimate the statistical heterogeneity, the Higgins and Thompson I-square (*I*^2^) was used (25). Heterogeneity was considered unimportant when the I-square values were on the order of 0% to 40%, moderate when the percentages were 30% to 60%, substantial when *I*^2^ was 50% to 90% and very substantial when values were 75% to 100% (26). The estimated met analytic association measure was the odds ratio, considering the respective 95% confidence intervals. Additionally, for the quantitative outcomes, the standardized mean difference was estimated. The model used was random effects using the restricted likelihood ratio and Der Simonian-Laird techniques (27). It was not possible to perform a sensitivity analysis due to the insufficient number of studies for this analysis.

## Results

### Selection of studies

**Figure 1:**
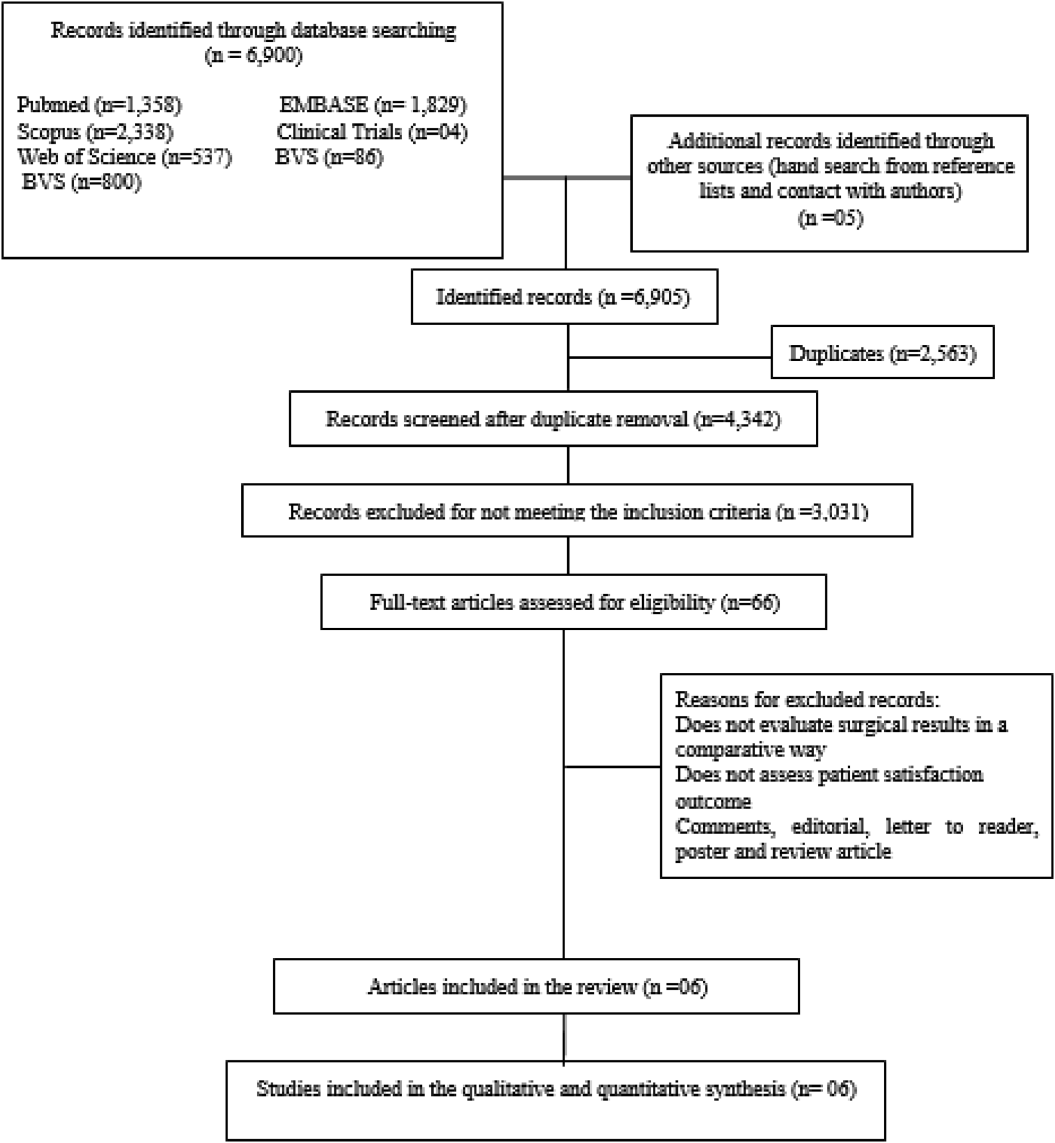
Flowchart of the study selection process.

A total of 6,900 studies were retrieved from searches in the databases, and 5 more studies were obtained by manual search of the reference lists of other articles. After the removal of duplicate records and exclusion by eligibility criteria (Figure 1), 3,031 articles were evaluated for selection of titles and abstracts. A total of 66 full-text articles were evaluated and sent for full reading. Finally, 6 studies were included in the qualitative and quantitative analysis, with a total of 1,182 patients. The characteristics of the studies are described in Table 1 (Characteristics of the included studies).

**Table 1:** Characteristics of the included studies.

Author	Period	Country	Study	Sample (*N*)	Follow-up (months)	Questionnaire
Ojala et al. (28)	2010	Finland	Retrospective cohort	379	36	BCTOS Questionnaire created by the author
Massa et al. (29)	2004	Italy	Prospective cohort	96	Group 1–62 Group 2–35 Group 3–60	Questionnaire created by the author
Santos et al. (30)	2007–2012	Brazil	Retrospective cohort	122	BCS 36.2 +/− 19.8 OPS 40.8 +/−16.3	BCCT.core software
Eichler et al. (8)	2007	Germany	Retrospective cohort	143	–	Questionnaire created by the author
de Oliveira-Júnior et al. (31)	May 2015—June, 2016	Brazil	Prospective cohort	300	7.14 (6.60–7.68)	EORTC QLQ-C30—EORTC QLQ BR 23 BCTOS QLQ
Tenofsky et al. (5)	April—December 2016	United States	Retrospective cohort	142	BCS 26.2 +/=16,5 OPS 24,6 +/− 10,2	Medical record information

Group 1—oncoplastic + external radiotherapy.

Group 2—oncoplastic + IORT (intraoperative radiation therapy).

Group 3—conventional + external radiotherapy.

BCS, breast conservative surgery; OPS, oncoplastic surgery; BCTOS, breast cancer treatment outcome scale; BCCT.core software, breast cancer conservative treatment cosmetic results software; EORTC QLQ-C30, European organization for research and treatment of cancer quality of life questionnaire; EORTC QLQ BR 23, European organization for research and treatment of cancer breast cancer -specific quality of life questionnaire; BCTOS QLQ, portuguese/Brazil version of breast cancer treatment outcome scale quality of life questionnaire.

### Characteristics of the study and participants

The sample population was composed exclusively of women, and the average age of the participants, when subjected to surgical treatment, was 57.71 years in the oncoplastic group (25.8–92 years) and 58.8 (32.8–91) years in the surgery group without oncoplastic surgery. Of the 1,182 people who underwent surgery and who answered the questionnaires, 31 (89%) underwent OPS, and the remainder underwent BCS. The mean follow-up of the studies was 31.4 months in the oncoplastic surgery group and 33.2 months in the Non oncoplastic surgery group.

### Aesthetic outcome

**Figure 2:**
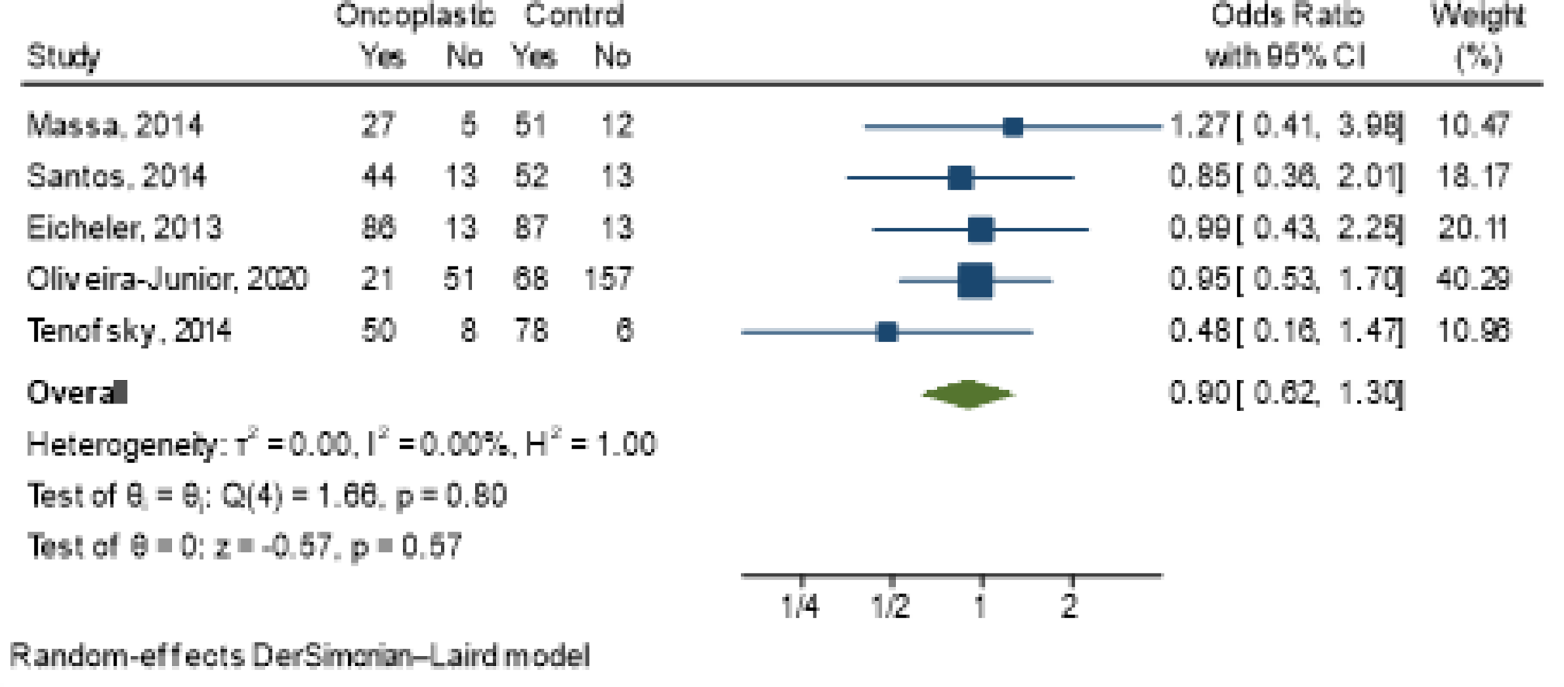
Meta-analysis of the outcome satisfaction with the type of surgery performed with or without oncoplastic surgery.

There was no difference in the degree of satisfaction of the participants between the conventional and oncoplastic conservative surgeries (HR 0.90; 95% CI 0.62–1.30), and low heterogeneity in the studies was observed with *I*^2^ = 0.00 (Figure 2). However, oncoplastic surgery allows patient satisfaction rates to be maintained even in the case of large or multicentric tumors, demonstrating the benefit of this surgical modality.

**Figure 3:**
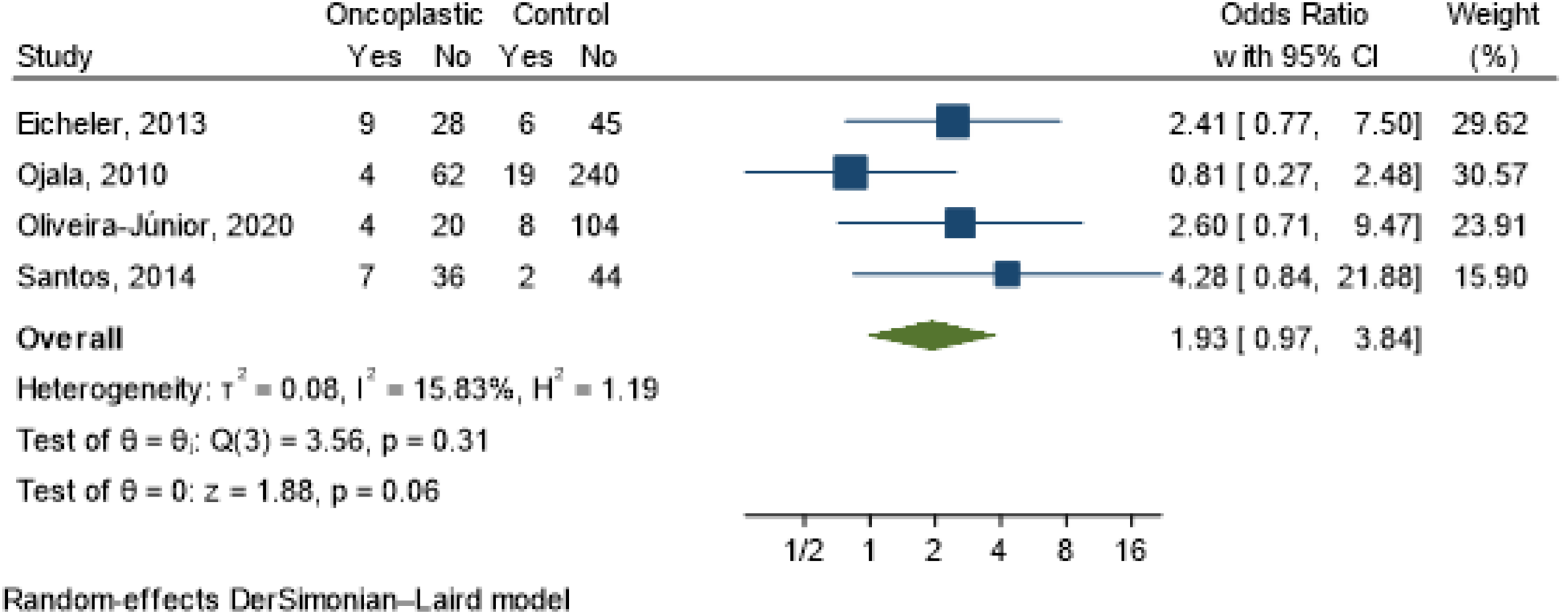
Meta-analysis for staging in conservative surgery with and without oncoplastic.

**Figure 4:**
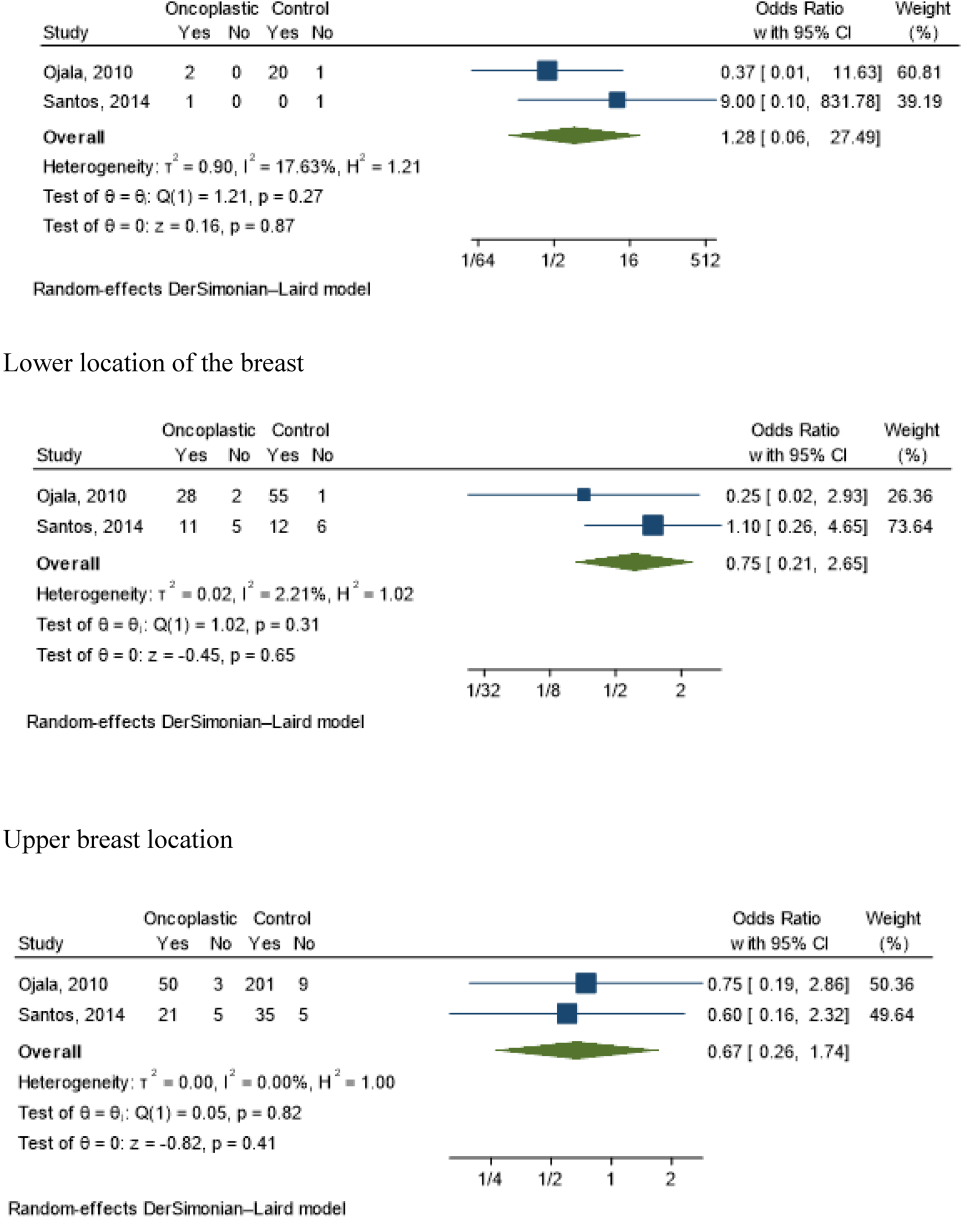
Meta-analyses for tumor localization in conservative surgery with and without oncoplastic. Central location of the breast. Lower location of the breast. Upper breast location.

**Figure 5:**
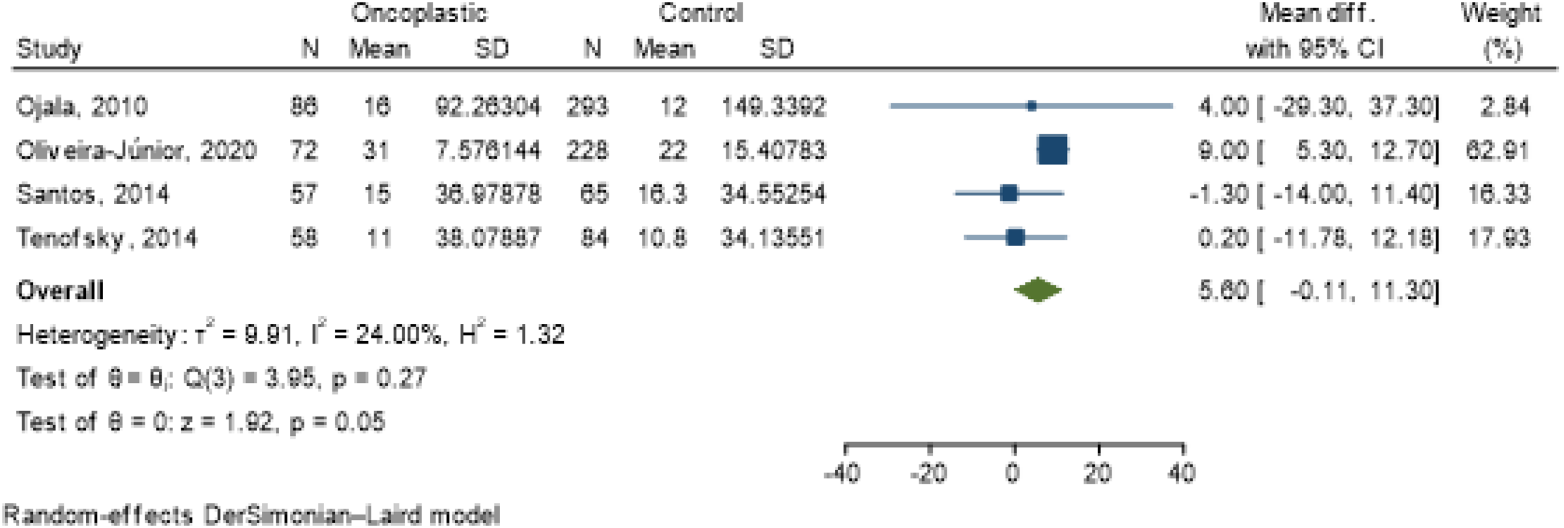
Meta-analysis for tumor size in conservative surgery with and without oncoplastic.

Staging (OR 1.93; 95% CI 0.97–3.84; *I*^2^ = 15.83%) (Figure 3); tumor location [central (OR 1.28; 95% CI 0.06–27.49; *I*^2^ = 17.63%); lower (OR 0.75; 95% CI 0.21–2.65; *I*^2^ = 2.21%); superior (OR 0.67; 95% CI 0.26–1.74; *I*^2^ = 0.00%] (Figure 4) and tumor size (OR 5.6; 95% CI −0.11–11.30; *I*^2^ = 24%) (Figure 5) showed no association with the type of conservative surgery performed, with or without oncoplastic surgery. The outcome was dichotomized into excellent/good and fair/poor, and despite the limitations of this form of evaluation, it remains the most used method (15).

### Type of surgery performed

Several different oncoplastic surgery techniques were used, and the number of patients in each of them was small. Only three articles clearly described the surgical modalities used (Table 2).

**Table 2:** Specification of the surgical technique used.

Study/sample (*N*)	Technique used
Ojala et al. (28) *N* 86	Racket mammoplasty 19 (22%) Reduction mammoplasty techniques 19 (22%) Round block 16 (19%) Rotationplasty techniques 16 (19%) Extensive dual plane undermining 12 (14%) Other oncoplastic techniques 4 (5%)
Oliveira-Júnior et al. (31) *N* 72	Pedicle surgery 34 (47.2%) Central plug-flap procedure 8 (11.1%) Dermoglandular flap or a local flap procedure 12 (16.7%) Periareolar procedure 7 (9.7%) Reconstruction with the latissimus dorsi muscle 6 (8.3%) Remodeling 2 (2.8%) Fat grafting (lipofilling) 2 (2.8%) Breast implant 1 (1.4%)
Tenofsky et al. (5) *N* 58	Therapeutic mammoplasty 14 (24%) Adjacent tissue transfers 43 (74%) Donut mastopexy 5 (7%) Some of these patients received a mixture of multiple oncoplastic techniques.

### Questionnaires used

Each study used different questionnaires with different evaluation criteria, some of which were standardized and established in the literature, but other specific questionnaires were created exclusively for the study in question (Tables 1, 3).

**Table 3:** Evaluation criteria of the questionnaires used.

Results	Excellent/good/sufficient^#^		Intermediate		Poor/insufficient		Comments	Parameters analyzed
Studies	Conservative	Oncoplastic	Conservative	Oncoplastic	Conservative	Oncoplastic		
Ojala et al. (28) *N* 379	BCTOS Total 284 (75%)		–		–		The mean aesthetic score of the BCTOS was worse after oncoplastic resection than the conventional resection (mean 1.84 × 1.62; *p* = 0.002)	Aesthetic status, functional status, and sensitivity
	230 (81%) *N* 293	52 (61%) *N* 86						
	Questionnaire created by the author		–		–		The position and appearance of the nipple, the breast scar and the excellent result were significantly better in the conventional resection group. Regarding breast size, position and shape of the breast, there were no significant differences between the groups.	Breast size, appearance and position of the nipple, shape and position of the breast, scar tissue, bra adjustment and symmetry of the contralateral breast
	217 (75%)	61 (72%)						
Massa et al. (29) *N* 96	Group 3–51 (81%) *N* 64	Group 1–12 (75%) Group 2–15 (93,75%) *N* 32	–		Group 3–12 (19%)	Group 1–4 (25%) Group 2–1 (6,25%)	Regarding the aesthetic parameters, all patients in the 3 groups expressed a favorable judgment (scores ≥6)	Breast symmetry, scar, areopapillary complex symmetry and overall judgment
Santos et al. (30) *N* 122	**BCCT.core** Excellent 4 (6.2%) Good 48 (73.8%) *N* 65	Excellent 13 (22.8%) Good 31 (54.4%) *N* 57	Regular 10 (15.4%)	Regular 12 (21.1%)	Poor 3 (4.6%)	Poor 1 (1.8%)	**BCCT.core software:** an algorithm summarizes all objective symmetry measurements. The results are based on a four -point scale: excellent, good, fair, and poor. **Experts:** The conclusions were graded from 0 to 10. Zero corresponded to the worst and 10 to the best result. **Patients:** answered a questionnaire about their satisfaction with the aesthetic results with answers classified as excellent, good, fair, and unsatisfactory	**BCCT.core software:** Points designated by the examiner and automated software calculations of different measures of symmetry, breast volume, skin color and scars **Experts:** volume, shape, mammary symmetry, position of the inframammary fold and scars **Patients:** satisfaction with aesthetic results
	**Experts** Excellent 12 (18.5%) Good 40 (61.5)	Excellent 29 (50.9%) Good 23 (40.4%)	Regular 12 (18.5%)	Regular 4 (7.0%)	Poor 1 (1.5%)	Poor 1 (1.8%)		
	**Patients** Excellent 45 (69.2) Good 19 (29.2)	Excellent 35 (61.4) Bom 17 (29.8)	Regular 1 (1.5%)	Regular 2 (3.5%)	Poor 0 (0.0)	Poor 3 (5.3)		
Eichler et al. (8) *N* 143	*N* 71	*N* 72	Evaluation by item and not global		–		–	(1) Evaluate the overall cosmetic outcome of your breast. (2) Are you satisfied with the appearance and amount of scar tissue? (3) Do you like the current shape of the breast? (4) Are you currently satisfied with the appearance of the breast? (5) Are you currently satisfied with your breast size? (6) Evaluate your current quality of life. (7) Has the sensitivity of the nipple/areolar complex changed, increased/decreased? (8) Was there a significant amount of swelling in and around the breast area? (9) Is it less likely that you show yourself in public? (10) Has your level of self -confidence changed due to surgery?
de Oliveira-Júnior et al. (31) *N* 300	Excelente 13 (72.2%) Bom 55 (77.5%) *N* 228	Excelente 5 (27.8%) Bom 16 (22.5%) *N* 72	Regular 97 (71.9%)	Regular 38 (28.1%)	Poor 60 (82.2%)	Poor 13 (17.8%)	The patients were submitted to the QLQ, EORTC QLQC30, EORTC QLQ-BR23 and BCTOS, which were combined with a clinical visit. The data from the medical records were retrospectively evaluated using standardized records. The BCTOS contains 22 questions divided into 4 domains (functional, cosmetic, pain and edema); the cosmetic domain is composed of 8 questions. The questions compare the surgical results of the treated breast versus the untreated breast on a scale of 1 to 4 (1 = no difference, 4 = significant difference).	**EORTC QLQC30:** Global health, Physical, functional, emotional, cognitive, social capacity, Fatigue, Nausea and vomiting, Pain, Dyspnea, Insomnia, Loss of appetite, Constipation, Diarrhea, Financial difficulties **EORTC QLQ-BR23:** Body image, Sexual functioning, Sexual pleasure, Future perspective, Side effects, Breast symptoms, Arm symptoms, Hair loss **BCTOS:** Functional, Cosmetic, Breast pain, Edema
Tenofsky et al. (5) 142	78 (92%) *N* 84	50 (86%) *N* 58	–		6 (7.1%)	8 (13.8%)	Most patients were satisfied with the cosmetic result reported subjectively by the patient	Subjective report

^#^Represents only a highlight.

### Evaluation of study quality and risk of bias

The quality assessment for each study is shown in Table 4. All studies appeared to have good/excellent methodological quality, with a mean of 9 “yes” answers.

**Table 4:** Critical evaluation of the joanna briggs institute for cohort studies.

Questions	Ojala, 2010	Massa, 2014	Santos, 2014	Eichler et al. (8)	de Oliveira-Junior, 2020	Tenofsky et al. (5)
1. Were the two groups similar and recruited from the same population?	Y	Y	Y	Y	Y	Y
2. Were the exposures measured similarly to assign people to both exposed and unexposed groups?	Y	Y	Y	Y	Y	Y
3. Was the exposure measured in a valid and reliable way?	Y	Y	Y	N	Y	Y
4. Were confounding factors identified?	Y	Y	Y	Y	Y	Y
5. Were strategies to address confounding factors stated?	Y	Y	Y	Y	Y	Y
6. Were the groups/participants free of the outcome at the start of the study (or at the moment of exposure)?	Y	Y	Y	Y	Y	Y
7. Were the outcomes measured in a valid and reliable way?	Y	Y	Y	Y	Y	Y
8. Was the follow up time reported and sufficient to be long enough for outcomes to occur?	Y	Y	Y	Y	Y	Y
9. Was follow up complete, and if not, were the reasons to loss to follow up described and explored?	Y	U	Y	Y	Y	Y
10. Were strategies to address incomplete follow up utilized?	U	U	Y	U	Y	U
11. Was appropriate statistical analysis used?	U	Y	Y	U	Y	Y
Total number of “y” answers	9	9	11	8	11	10

Y, yes; U, unclear.

The Newcastle‒Ottawa Quality Assessment Form for Cohort Studies tool was used to assess the risk of bias, and the results revealed that the studies had good and reasonable quality, demonstrating a reasonable compromise in the representativeness domain of patient selection, as shown in Table 5.

**Table 5:** Risk of bias by the Newcastle‒Ottawa quality assessment for cohort studies.

Authors	Study	Selection	Comparability	Outcome	Follow -up	Adequacy	Total
Ojala et al. (28)	Retrospective cohort	4	1	1	1	1	8 Excellent quality
Massa et al. (29)	Prospective cohort	2	1	1	1	1	6 Reasonable quality
Santos et al. (30)	Retrospective cohort	2	1	1	1	1	6 Reasonable quality
Eichler et al. (8)	Retrospective cohort	4	1	1	1	0	8 Excellent quality
Oliveira-júnior et al. (31)	Prospective cohort	4	1	1	1	1	8 Excellent quality
Tenofsky et al. (5)	Retrospective cohort	4	1	1	1	1	8 Excellent quality

### Quality of evidence

According to the evaluation of the Grade System (23), the classification of evidence was of very low quality due to high inconsistency between studies, statistical inaccuracies and possibility of selection bias and, consequently, low recommendation strength (Table 6).

**Table 6:** GRADE of GRADE evidence quality.

Certainty of evidence						
Participants (studies) Follow -up	Risk of bias	Inconsistency	Indirect evidence	Inaccuracy	Publication bias	Overall certainty of evidence
Tumor location—Central (2 observational studies)	Severe	Non -severe	Non -severe	Severe	Highly suspicious publication biases all potential confounding factors would suggest a spurious effect and, even so, no effect was observed.	⊕ ◯◯◯ Very low
Tumor location—Lower (2 observational studies)	Severe	Non -severe	Non -severe	Non -severe	Highly suspicious publication biases all potential confounding factors would suggest a spurious effect and, even so, no effect was observed.	⊕ ◯◯◯ Very low
Tumor location—Upper (2 observational studies)	Severe	Non -severe	Non -severe	Non -severe	Highly suspicious publication biases all potential confounding factors would suggest a spurious effect and, even so, no effect was observed.	⊕ ◯◯◯ Very low
Staging (4 observational studies)	Severe	Non -severe	Non -severe	Non -severe	Highly suspicious publication biases all potential confounding factors would suggest a spurious effect and, even so, no effect was observed.	⊕ ◯◯◯ Very low
Aesthetic result (5 observational studies)	Severe	Non -severe	Non -severe	Non -severe	Highly suspicious publication biases all potential confounding factors would suggest a spurious effect and, even so, no effect was observed.	⊕ ◯◯◯ Very low
Tumor size (4 observational studies)	Severe	Very severe	Non -severe	Severe	Highly suspicious publication biases all potential confounding factors would suggest a spurious effect and, even so, no effect was observed.	⊕ ◯◯◯ Very low

## Discussion

The reasons for recommending oncoplastic surgery are usually associated with cosmesis. Our study compared satisfaction with the aesthetic result among women undergoing BCS, with or without OPS, and demonstrated no effect of the treatment. We highlight, however, that oncoplasty allows patient satisfaction rates to be maintained even in the case of large or multicentric tumors when radical techniques would be more indicated. Maintaining satisfaction even in unfavorable contexts to maintain the aesthetic result.

There was excellent/good patient satisfaction with surgical treatment in both types of conservative surgery, with more than 60% of women reporting it (4). Breast symmetry (32), the appearance of the residual scar, the symmetry between the 2 nipple-areolar complexes, the overall aesthetic judgment and satisfaction with the result (4), and the change in the appearance of the breast (33) are the factors most likely to be evaluated in the aesthetic judgment of the operated breast.

Oncoplastic surgeries have recently become an option for the surgical treatment of breast cancer, but the procedures require additional time (16) and specialized training (34) and, in general, can be technically and surgically demanding (3, 6, 35). The aesthetic result may be affected by the indication of the specific surgical technique (36), the surgeon's experience and their learning curve (5). Mastopexy can be offered as an option when simultaneous reduction of the breasts is desired, with low postoperative complications and good subjective patient satisfaction in the postoperative period (8, 37). BCS involves a shorter surgical time (16) and leads to less formation of scar tissue, although this does not significantly influence the aesthetic parameter (8). The aesthetic result and immediate complications are similar without the latter leading to delayed radiotherapy (1, 2, 16, 36) and the exception of wounds that do not heal and the occurrence of fat necrosis, which are more common in the oncoplastic group (5). The excision rate seems similar between the techniques and is more related to tumor histological factors (1). The two surgical options are effective and well indicated depending on the individuality of each case.

Data from the literature show that patients with oncoplastic surgery had a lower percentage of positive margins and a lower rescission rate (3). In addition, they were younger than conventional surgery patients (1), more frequently in the premenopausal period, as well as presenting greater weight of the surgical specimen (32), more advanced staging, larger tumor size (21, 26, 38), and components of extensive ductal carcinoma *in situ* (39), and this fact is related to the worst tumor conditions related to oncoplastic surgeries (1, 3, 15), unfavorable biology (16), more tumors in the inferior retroareolar region, a higher probability of receiving neoadjuvant chemotherapy (2, 3, 16) and multifocality appearing to be these last three factors associated with dissatisfaction with the aesthetic result (14). These data are not corroborated in the present study and were also present in another meta-analysis, and satisfaction with the aesthetic result was significantly higher in the oncoplastic group (32) showing that this comparison still presents controversial results. Interestingly, tumor location, radiotherapy and tumor bed reinforcement had no effect on aesthetics (14).

Breast symmetry was not associated with high patient satisfaction, and bilateral surgery (breast symmetrization) was not associated with changes in quality of life (3). The patients undergoing oncoplastic surgery were younger and had a higher level of education, which may have increased the degree of demand for better aesthetic results. In addition, they had larger tumors and at a more advanced stage, which could be a contraindication to classical conservative surgery (38). This makes the selection bias evident (8). Individuals with a high number of comorbidities and elderly women do not usually suffer symmetrization (3).

In these situations, in which conservative treatment would bring unacceptable sequelae and in which mastectomy would be the most common option (extreme oncoplasty), we believe that the fairest comparison is not between classic conservative treatment and oncoplastic surgery but between oncoplastic surgery and mastectomy with total breast reconstruction.

Most patients undergoing conservative surgery do not require reconstruction or posterior repair unless severe breast damage is caused after surgery (8, 39). The degree of satisfaction does not necessarily reflect the degree of symmetry (4). Satisfaction with the surgical outcome is affected by the time elapsed from surgery to the measurement of the outcome because it is known that time changes a person's body image and the perception of their own body (3). In addition, adaptation with the new self-image can bring satisfaction even in the face of a Non aesthetically acceptable result, which further reflects the psychosocial adaptation of patients with the new self-image (15) associated with the relief of tumor resection and removal of the disease. Women with normal breasts may be dissatisfied with their breast aesthetics, and patients with high expectations may have less satisfaction, even with good to excellent results (4). The evaluation of patients is more related to quality of life issues than just the aesthetic characteristics (15, 38), and patient dissatisfaction may be correlated with postoperative complications and breast asymmetry (32).

It is possible that patient satisfaction was influenced by the degree of expectation of patients (40). That is, women who underwent classical conservative surgery might have had lower expectations of good outcomes and could have been satisfied even with worse outcomes. Similarly, women undergoing oncoplastic surgery could have had a high expectation, similar to that of cosmetic surgery. We know that tumor repair can be difficult and not always total, even with oncoplastic surgery. In addition, there are deleterious effects of radiotherapy. Thus, small asymmetries and tolerable imperfections in conservative treatment may have negatively impacted the satisfaction of women undergoing oncoplastic surgery, even if the result was better.

No specific questionnaire was developed for the evaluation of oncoplastic surgery, representing a significant gap in the literature that needs to be filled. Studies evaluating the quality of life of patients undergoing oncoplastic surgery are limited, and the results are controversial considering the diversity of procedures (3). There is no consensus on the best instrument to use, the best way to collect the data or how to interpret the results were not established in a standard way (3). The comparative analysis of the results evaluated using objective instruments is extraordinarily complex and may not be reproducible, making comparison with subjective evaluations even more infeasible. A study comparing objective data evaluated using the BCCT.core software and self-reported patient data showed that patients tended to be more satisfied in the personal assessment with subjective parameters than that observed in the objective assessment (3, 4). Most studies are retrospective series, editorials and review articles (level III/V of evidence). There is subsequently a lack of randomized control data comparing the two groups (conservative with or without oncoplastic).

The oncoplastic techniques applied were diverse in relation to the various articles, with little standardization (34, 36, 41) which hinders the scientific comparison of the techniques among themselves (16). Some studies have reported surgical indication more generally but following a principle according to the description of oncoplastic techniques proposed by the French surgeon Krishna Clough (36), which classifies surgery as level I (excision of less than 20% of the breast volume during conservative surgery in small to moderate breasts with minimal ptosis) and level II (excision of 20% to 50% of the breast tissue in breast size) moderate to large with moderate to severe ptosis) (4, 6, 42). The group of conservative surgery without oncoplastic was subjected to incisions on the tumor without skin removal (except in cases where the tumors were close to the skin) (6) or periareolar incisions (8). One study showed that in the oncoplastic resection group, there was a significant difference in the aesthetic result between the techniques, with the dual plane having the worst aesthetic result and the reduction mammoplasty and round block techniques showing the best aesthetic results (14).

The reduction of breast size using oncoplastic techniques can bring benefits by minimizing the undesirable effects of radiation, such as chronic pain, radiation toxicity to the skin, vasculitis, and fibrosis of the breast parenchyma, which are more intense in larger breast volumes. In addition, it does not seem to negatively affect the duration of treatment and the occurrence of recurrence (5). In addition, innovations in radiotherapy with the introduction of partial breast irradiation techniques also have a strong focus on aesthetic results that need to be compared with those of whole-breast irradiation. Thus, with the new oncoplastic and radiotherapy interventions, there will be even more demand for the evaluation of the aesthetic result (41) and with this the need to fill the gap left by the absence of a gold standard (17, 32) that can define a set of recommendations to be used in clinical practice to better measure the cosmetic outcome (15, 36).

The main limitation of the present study was the selection bias favoring the conventional resection group. Most studies are retrospective with a design that does not allow the determination of risk factors (1). The number of patients in the oncoplastic surgery group was also lower. Several oncoplastic techniques were used, and the number of patients in each technique used was small. Therefore, some significant associations may have remained undetected. A likely assumption would be that some studies may have collected data from the period in which the oncoplastic age coincided with the onset of services and that breast surgeons were still at the beginning of their learning curve, which may interfere with the final aesthetic outcome. Although extensive research was conducted in several databases, there was only a limited amount of evidence available on the subject. Thus, it was not possible to apply other more robust analytical techniques, such as evaluation of publication bias, Egger's test, subgroup analysis, sensitivity analysis and meta-regressions. A possible source of heterogeneity may have been the inclusion of methodologically different studies, diversity in the use of the outcome measurement questionnaire, insufficient record of information in the original study, regional differences, and use of various combined surgical techniques. The evaluation of the grade indicated an extremely low quality of evidence due to the risk of bias, inaccuracy, and inconsistency. Thus, the results of this review should be carefully evaluated before being considered as a recommendation.

This meta-analysis used validated instruments for the sensitive evaluation of search strategies, measurement of methodological quality and writing of systematic reviews, such as PRESS, Joanna Briggs, MOOSE and Prisma, respectively, in addition to the risk of bias with the Newcastle-Ottawa Scale. Another positive aspect was the selection of cohorts, with a safe record of the intervention and outcome, minimizing the possibility of information bias. In the absence of randomized prospective studies, this systematic review with meta-analysis is the main scientific evidence on the subject, and it is the only one that has been registered in Pros to date on the subject. The individual sample of each study included in this study was small; however, when performing the analysis together in the meta-analysis, the sample size was significant and with good comparability between groups, which helped to minimize the evaluation distortions (8).

The assessment of satisfaction is subjective and may not be exactly related to the aesthetic result but to the acceptance of the new self-image. A satisfied patient does not necessarily have a more beautiful and symmetrical breast than another dissatisfied patient. The beauty in terms of symmetry and greater similarity with a natural breast should be evaluated by objective parameters by the patient herself, a blind observer, and the surgeon. These results of similarity of satisfaction in the two groups do not represent that the aesthetic result of conventional surgery is similar to that of oncoplastic surgery but rather that patient satisfaction may be similar after a considerable time from surgery to evaluation (38) which allows an adaptation to the new self-image. Thus, a new evaluation in terms of beauty and symmetry would be interesting, which would probably show the superiority of oncoplastic surgery because, despite being a procedure with longer surgical time and greater number of scars, there are no differences in complications and cancer safety, allowing greater resection with wider margins and adding a better aesthetic result. It is also noteworthy that most studies are based on a retrospective analysis, with its inherent bias, and there are no clear criteria defining which patients should undergo oncoplastic surgery.

The review found that the current evidence base is limited, inadequate and robust enough to support or reject the assumption that oncoplastic surgery is associated with improved quality of life when compared to conventional conservative surgery. However, most studies show that oncoplastic surgery was associated with a trend toward better quality of life of the patient but needs to be investigated with greater robustness, and prospective studies of high methodological quality are strongly recommended (43).

Regarding the future, the COSMAM Trial (44) aims to prospectively perform and determine the clinical value of different techniques in breast-conserving surgery with regard to quality of life and aesthetic outcome. The analysis will be performed by objective measurements of the final cosmetic result compared to standard breast-conserving surgery. The results of this study will be used to develop a clinical decision model to guide the use of oncoplastic surgery in the future (44).

## Conclusion

The available evidence from the study indicated that oncoplastic surgery obtained a satisfaction with the results similar to that of conventional conservative surgery, even with a higher proportion of larger tumors, requiring greater resections. Extreme oncoplasty brings satisfactory results even in situations where surgical radicality and impaired aesthetic results were expected in the face of large tumors. From the aesthetic point of view, both oncoplastic and conventional treatment determine satisfactory aesthetic results; they should be considered safe alternatives for selected patients with breast cancer, and the indication of each technique will depend on the individuality of each case and the option of the patient herself and the conditions and experience of the medical service.

## Data Availability

The original contributions presented in the study are included in the article/**Supplementary Material**, further inquiries can be directed to the corresponding author.

## References

[1] Acea-Nebril B Cereijo-Garea C García-Novoa A Varela-Lamas C Builes-Ramírez S Bouzón-Alejandro A The role of oncoplastic breast reduction in the conservative management of breast cancer: complications, survival, and quality of life. J Surg Oncol. (2017) 115(6):679–86. 10.1002/jso.24550 28083875

[2] Wijgman DJ Ten Wolde B van Groesen NRA Keemers-Gels ME van den Wildenberg FJH Strobbe LJA. Short term safety of oncoplastic breast conserving surgery for larger tumors. Eur J Surg Oncol. (2017) 43(4):665–71. 10.1016/j.ejso.2016.11.021 28041648

[3] de Oliveira-Junior I da Silva IDA da Silva FCB da Silva JJ Sarri AJ Paiva CE Oncoplastic surgery in breast-conserving treatment: patient profile and impact on quality of life. Breast Care Basel Switz. (2021) 16(3):243–53. 10.1159/000507240 PMC8248771 34248465

[4] Massa M Meszaros P Baldelli I Bisso N Franchelli S. Aesthetic evaluation in oncoplastic and conservative breast surgery: a comparative analysis. Plast Reconstr Surg Glob Open. (2015) 3(3):e339. 10.1097/GOX.0000000000000309 26034646 PMC4448714

[5] Tenofsky PL Dowell P Topalovski T Helmer SD. Surgical, oncologic, and cosmetic differences between oncoplastic and nononcoplastic breast conserving surgery in breast cancer patients. Am J Surg. (2014) 207(3):398–402; discussion 402. 10.1016/j.amjsurg.2013.09.017 24581764

[6] Santos G Urban C Edelweiss MI Zucca-Matthes G de Oliveira VM Arana GH Long-Term comparison of aesthetical outcomes after oncoplastic surgery and lumpectomy in breast cancer patients. Ann Surg Oncol. (2015) 22(8):2500–8. 10.1245/s10434-014-4301-6 25519931

[7] Kosasih S Tayeh S Mokbel K Kasem A. Is oncoplastic breast conserving surgery oncologically safe? A meta-analysis of 18,103 patients. Am J Surg. (2020) 220(2):385–92. 10.1016/j.amjsurg.2019.12.019 31926592

[8] Eichler C Kolsch M Sauerwald A Bach A Gluz O Warm M. Lumpectomy versus mastopexy–a post-surgery patient survey. Anticancer Res. (2013) 33(2):731–6. PMID: 23393375 23393375

[9] Han J Grothuesmann D Neises M Hille U Hillemanns P. Quality of life and satisfaction after breast cancer operation. Arch Gynecol Obstet. (2010) 282(1):75–82. 10.1007/s00404-009-1302-y 19960349

[10] Mascaro A Farina M Gigli R Vitelli CE Fortunato L. Recent advances in the surgical care of breast cancer patients. World J Surg Oncol. (2010) 8(1):5. 10.1186/1477-7819-8-5 20089167 PMC2828445

[11] Carriero S Lanza C Pellegrino G Ascenti V Sattin C Pizzi C Ablative therapies for breast cancer: state of art. Technol Cancer Res Treat. (2023) 22:15330338231157193. 10.1177/15330338231157193 36916200 PMC10017926

[12] Silverstein MJ. Radical mastectomy to radical conservation (extreme oncoplasty): a revolutionary change. J Am Coll Surg. (2016) 222(1):1–9. 10.1016/j.jamcollsurg.2015.10.007 26778582

[13] Savioli F Seth S Morrow E Doughty J Stallard S Malyon A Extreme oncoplasty: breast conservation in patients with large, multifocal, and multicentric breast cancer. Breast Cancer Dove Med Press. (2021) 13:353–9. PMID: 34079367 34079367 10.2147/BCTT.S296242 PMC8164874

[14] Ojala K Meretoja TJ Leidenius MHK. Aesthetic and functional outcome after breast conserving surgery—comparison between conventional and oncoplastic resection. Eur J Surg Oncol. (2017) 43(4):658–64. 10.1016/j.ejso.2016.11.019 28040314

[15] Cardoso MJ Cardoso JS Vrieling C Macmillan D Rainsbury D Heil J Recommendations for the aesthetic evaluation of breast cancer conservative treatment. Breast Cancer Res Treat. (2012) 135(3):629–37. 10.1007/s10549-012-1978-8 22307267

[16] Kelemen P Pukancsik D Újhelyi M Sávolt Á Kovács E Ivády G Comparison of clinicopathologic, cosmetic and quality of life outcomes in 700 oncoplastic and conventional breast-conserving surgery cases: a single-centre retrospective study. Eur J Surg Oncol. (2019) 45(2):118–24. 10.1016/j.ejso.2018.09.006 30352766

[17] Zehra S Doyle F Barry M Walsh S Kell MR. Health-related quality of life following breast reconstruction compared to total mastectomy and breast-conserving surgery among breast cancer survivors: a systematic review and meta-analysis. Breast Cancer. (2020) 27(4):534–66. 10.1007/s12282-020-01076-1 32162181

[18] Stroup DF Berlin JA Morton SC Olkin I Williamson GD Rennie D Meta-analysis of observational studies in epidemiology: a proposal for reporting. Meta-analysis of observational studies in epidemiology (MOOSE) group. JAMA. (2000) 283(15):2008–12. 10.1001/jama.283.15.2008 10789670

[19] Moher D Liberati A Tetzlaff J Altman DG, PRISMA Group. Preferred reporting items for systematic reviews and meta-analyses: the PRISMA statement. PLoS Med. (2009) 6(7):e1000097. 10.1371/journal.pmed.1000097 19621072 PMC2707599

[20] McGowan J Sampson M Salzwedel DM Cogo E Foerster V Lefebvre C. PRESS Peer review of electronic search strategies: 2015 guideline statement. J Clin Epidemiol. (2016) 75:40–6. 10.1016/j.jclinepi.2016.01.021 27005575

[21] Ouzzani M Hammady H Fedorowicz Z Elmagarmid A. Rayyan-a web, and mobile app for systematic reviews. Syst Rev. (2016) 5(1):210. 10.1186/s13643-016-0384-4 27919275 PMC5139140

[22] Martin J. © Joanna Briggs Institute 2017 Critical Appraisal Checklist for Cohort Studies. Australia: University of Adelaide (2017). p. 7. Available online at: https://jbi.global/sites/default/files/2019-05/JBI_Critical_Appraisal-Checklist_for_Cohort_Studies2017_0.pdf (Accessed May 22, 2022).

[23] GRADEpro. Disponível em: Available online at: https://www.gradepro.org/ (Accessed May 6, 2022).

[24] Gierisch JM Beadles C Shapiro A McDuffie JR Cunningham N Bradford D Newcastle-Ottawa Scale Coding Manual for Cohort Studies. Health Disparities in Quality Indicators of Healthcare Among Adults with Mental Illness. Washington, DC: Department of Veterans Affairs (US) (2014). Disponível em: https://www.ncbi.nlm.nih.gov/books/NBK299087/ (citado de maio de 20, 2022). 26065051

[25] Higgins JPT Thompson SG Deeks JJ Altman DG. Measuring inconsistency in meta-analyses. Br Med J. (2003) 327(7414):557–60. 10.1136/bmj.327.7414.557 12958120 PMC192859

[26] Higgins JPT Thomas J Chandler J Cumpston M Li T Page MJ, editors. Cochrane Handbook for Systematic Reviews of Interventions. 2nd ed. Chichester, UK: John Wiley & Sons (2019).

[27] DerSimonian R Laird N. Meta-analysis in clinical trials revisited. Contemp Clin Trials. (2015) 45(Pt A):139–45. 10.1016/j.cct.2015.09.002 26343745 PMC4639420

[28] Ojala K Meretoja TJ Leidenius MHK. Aesthetic and functional outcome after breast conserving surgery—comparison between conventional and oncoplastic resection. Eur J Surg Oncol. (2017) 43(4):658–64. 10.1016/j.ejso.2016.11.019 28040314

[29] Massa M Meszaros P Baldelli I Bisso N Franchelli S. Aesthetic evaluation in oncoplastic and conservative breast surgery: a comparative analysis. Plast Reconstr Surg Glob Open. (2015) 3(3):e339. 10.1097/GOX.0000000000000309 26034646 PMC4448714

[30] Santos G Urban C Edelweiss MI Zucca-Matthes G de Oliveira VM Arana GH Long-Term comparison of aesthetical outcomes after oncoplastic surgery and lumpectomy in breast cancer patients. Ann Surg Oncol. (2015) 22(8):2500–8. 10.1245/s10434-014-4301-6 25519931

[31] de Oliveira-Junior I da Silva IDA da Silva FCB da Silva JJ Sarri AJ Paiva CE Oncoplastic surgery in breast-conserving treatment: patient profile and impact on quality of life. Breast Care Basel Switz. (2021) 16(3):243–53. 10.1159/000507240 PMC8248771 34248465

[32] Losken A Dugal CS Styblo TM Carlson GW. A meta-analysis comparing breast conservation therapy alone to the oncoplastic technique. Ann Plast Surg. (2014) 72(2):145–9. 10.1097/SAP.0b013e3182605598 23503430

[33] Shaitelman SF Jeruss JS Pusic AL. Oncoplastic surgery in the management of breast cancer. J Clin Oncol. (2020) 38(20):2246–53. 10.1200/JCO.19.02795 32442070

[34] Holmes D Schooler W Smith R. Oncoplastic approaches to breast conservation. Int J Breast Cancer. (2011) 2011:303879. 10.4061/2011/303879 22295216 PMC3262568

[35] Losken A Hart AM Chatterjee A. Updated evidence on the oncoplastic approach to breast conservation therapy. Plast Reconstr Surg. (2017) 140(5S Advances in Breast Reconstruction):14S–22S. 10.1097/PRS.0000000000003951 29064918

[36] Clough KB Kaufman GJ Nos C Buccimazza I Sarfati IM. Improving breast cancer surgery: a classification and quadrant per quadrant atlas for oncoplastic surgery. Ann Surg Oncol. (2010) 17(5):1375–91. 10.1245/s10434-009-0792-y 20140531

[37] Losken A Elwood ET Styblo TM Bostwick J. The role of reduction mammaplasty in reconstructing partial mastectomy defects. Plast Reconstr Surg. (2002) 109(3):968–75; discussion 976–977. 10.1097/00006534-200203000-00025 11884818

[38] Haloua MH Krekel NMA Winters HAH Rietveld DHF Meijer S Bloemers FW A systematic review of oncoplastic breast-conserving surgery current weaknesses and future prospects. Ann Surg. (2013) 257(4):609–20. 10.1097/SLA.0b013e3182888782 23470508

[39] Losken A Styblo TM Carlson GW Jones GE Amerson BJ. Management algorithm and outcome evaluation of partial mastectomy defects treated using reduction or mastopexy techniques. Ann Plast Surg. (2007) 59(3):235–42. 10.1097/SAP.0b013e31802ec6d1 17721207

[40] Spear SL Pelletiere CV Menon N. One-stage augmentation combined with mastopexy: aesthetic results and patient satisfaction. Aesthetic Plast Surg. (2004) 28(5):259–67. 10.1007/s00266-004-0032-6 15529204

[41] Clough KB Benyahi D Nos C Charles C Sarfati I. Oncoplastic surgery: pushing the limits of breast-conserving surgery. Breast J. (2015) 21(2):140–6. 10.1111/tbj.12372 25676776

[42] Cantürk NZ Şimşek T Özkan Gürdal S. Oncoplastic breast-conserving surgery according to tumor location. Eur J Breast Health. (2021) 17(3):220–33. 10.4274/ejbh.galenos.2021.2021-1-2 34263149 PMC8246052

[43] Aristokleous I Saddiq M. Quality of life after oncoplastic breast-conserving surgery: a systematic review. ANZ J Surg. (2019) 89(6):639–46. 10.1111/ans.15097 30977582

[44] Catsman CJLM Beek MA Voogd AC Mulder PGH Luiten EJT. The COSMAM TRIAL is a prospective cohort study of quality of life and cosmetic outcome in patients undergoing breast conserving surgery. BMC Cancer. (2018) 18(1):456. 10.1186/s12885-018-4368-8 29688847 PMC5914027

