# Supplementary material for: Comparative analysis of the degree of patient satisfaction after breast-conserving surgery with or without oncoplastic surgery: systematic review and meta-analysis
Source: Front Surg. 2024 Jul 16;11:1396432. doi: 10.3389/fsurg.2024.1396432 (PMC11289670; doi:10.3389/fsurg.2024.1396432)
Supplement: Supplementary file 1 [file Datasheet1.docx]

Appendix S1 - MOOSE Checklist for Meta-analyses of Observational Studies

| **Item No** | **Recommendation** | **Reported on Page No** |
| --- | --- | --- |
| Reporting of background should include | | |
| 1 | Problem definition | 4 |
| 2 | Hypothesis statement | 5 |
| 3 | Description of study outcome (s) | 5-6 |
| 4 | Type of exposure or intervention used | 5 |
| 5 | Type of study designs used | 6 |
| 6 | Study population | 6 |
| Reporting of search strategy should include | | |
| 7 | Qualifications of searchers (e.g., librarians and investigators) | 7 |
| 8 | Search strategy, including time period included in the synthesis and key words | 7 |
| 9 | Effort to include all available studies, including contact with authors | 7 |
| 10 | Databases and records searched | 7 |
| 11 | Search software used, name and version, including special features used (e.g., explosion) | 9 |
| 12 | Use of hand searching (e.g., reference lists of obtained articles) | 7 |
| 13 | List of citations located and those excluded, including justification | 24 |
| 14 | Method of addressing articles published in languages other than English | - - |
| 15 | Method of handling abstracts and unpublished studies | 24 |
| 16 | Description of any contact with authors | 7 |
| Reporting of methods should include | | |
| 17 | Description of relevance or appropriateness of studies assembled for assessing the hypothesis to be tested | 8 |
| 18 | Rationale for the selection and coding of data (e.g., sound clinical principles or convenience) | 6 |
| 19 | Documentation of how data were classified and coded (e.g., multiple raters, blinding and interrater reliability) | 8 |
| 20 | Assessment of confounding (e.g., comparability of cases and controls in studies where appropriate) | 9 |
| 21 | Assessment of study quality, including blinding of quality assessors, stratification or regression on possible predictors of study results | 8-9 |
| 22 | Assessment of heterogeneity | 9 |
| 23 | Description of statistical methods (e.g., complete description of fixed or random effects models, justification of whether the chosen models account for predictors of study results, dose‒response models, or cumulative meta-analysis) in sufficient detail to be replicated | 9 |
| 24 | Provision of appropriate tables and graphics | 24 -64 |
| Reporting of results should include | | |
| 25 | Graphic summarizing individual study estimates and overall estimate | 30-31 |
| 26 | Table giving descriptive information for each study included | 25 |
| 27 | Results of sensitivity testing (e.g., subgroup analysis) | - - |
| 28 | Indication of statistical uncertainty of findings | - - |

Appendix S2 - PRISMA checklist

| **Section/topic** | **#** | **Checklist item** | **Reported on page #** |
| --- | --- | --- | --- |
| **TITLE** | | |  |
| Title | 1 | Identify the report as a systematic review, meta-analysis, or both. | 01 |
| **ABSTRACT** | | |  |
| Structured summary | 2 | Provide a structured summary including as applicable: background; objectives; data sources; study eligibility criteria, participants, and interventions; study study appraisal and synthesis methods; results; limitations; conclusions and implications of key findings; systematic review registration number. | 2-3 |
| **INTRODUCTION** | | |  |
| Rationale | 3 | Describe the rationale for the review in the context of what is already known. | 4-5 |
| Objectives | 4 | Provide an explicit statement of questions being addressed with reference to participants, interventions, comparisons, outcomes, and study design (Picos). | 5-6 |
| **METHODS** | | |  |
| Protocol and registration | 5 | Indicate if a review protocol exists, if and where it can be accessed (e.g., Web address), and, if available, provide registration information including registration number. | 6 |
| Eligibility criteria | 6 | Specify study characteristics (e.g., Picos, length of follow-up) and report characteristics (e.g., years considered, language, publication status) used as criteria for eligibility, giving rationale. | 6 |
| Information sources | 7 | Describe all information sources (e.g., databases with dates of coverage, contact with study authors to identify additional studies) in the search and date last searched. | 7 |
| Search | 8 | Present full electronic search strategy for at least one database, including any limits used, such that it could be repeated. | 7 e 47-64 |
| Study selection | 9 | State the process for selecting studies (i.e., screening, eligibility, included in systematic review, and, if applicable, included in the meta-analysis). | 8 |
| Data collection process | 10 | Describe method of data extraction from reports (e.g., piloted forms, independently, in duplicate) and any processes for obtaining and confirming data from investigators. | 8 |
| Data items | 11 | List and defines all variables for which data were sought (e.g., Picos, funding sources) and any assumptions and simplifications made. | 7 |
| Risk of bias in individual studies | 12 | Describe methods used for assessing risk of bias of individual studies (including specification of whether this was done at the study or outcome level), and how this information is to be used in any data synthesis. | 8 |
| Summary measures | 13 | State the main summary measures (e.g., risk ratio, difference in means). | 9-10 |
| Synthesis of results | 14 | Describe the methods of handling data and combining results of studies, if done, including measures of consistency (e.g., I ^2^) for each meta-analysis. | 9-10 |

Page 1 of 2

| **Section/topic** | **#** | **Checklist item** | **Reported on page #** |
| --- | --- | --- | --- |
| Risk of bias across studies | 15 | Specify any assessment of risk of bias that may affect the cumulative evidence (e.g., publication bias, selective reporting within studies). | 9 |
| Additional analyzes | 16 | Describe methods of additional analyzes (e.g., sensitivity or subgroup analyzes, meta-regression), if done, indicating which were prespecified. | 9 |
| **RESULTS** | | |  |
| Study selection | 17 | Give numbers of screened studies, assessed for eligibility, and included in the review, with reasons for exclusions at each stage, ideally with a flow diagram. | 10 |
| Study characteristics | 18 | For each study, present characteristics for which data were extracted (e.g., study size, Picos, follow-up period) and provide the citations. | 10 |
| Risk of bias within studies | 19 | Present data on risk of bias of each study and, if available, any outcome level assessment (see item 12). | 12 |
| Results of individual studies | 20 | For all outcomes considered (benefits or harms), present, for each study: (a) simple summary data for each intervention group (b) effect estimates and confidence intervals, ideally with a forest plot. | 24 e 26 |
| Synthesis of results | 21 | Present results of each meta-analysis done, including confidence intervals and measures of consistency. | 11 |
| Risk of bias across studies | 22 | Present results of any assessment of risk of bias across studies (see Item 15). | 12 |
| Additional analysis | 23 | Give results of additional analyzes, if done (e.g., sensitivity or subgroup analyzes, meta-regression [see Item 16]). | 9 |
| **DISCUSSION** | | |  |
| Summary of evidence | 24 | Summarize the main findings including the strength of evidence for each main outcome; consider their relevance to key groups (e.g., healthcare providers, users, and policy makers). | 13 |
| Limitations | 25 | Discuss limitations at study and outcome level (e.g., risk of bias), and at review-level (e.g., incomplete retrieval of identified research, reporting bias). | 17 |
| Conclusions | 26 | Provide a general interpretation of the results in the context of other evidence, and implications for future research. | 19 |
| **FUNDING** | | |  |
| Funding | 27 | Describe sources of funding for the systematic review and other support (e.g., supply of data); role of funders for the systematic review. | 20 |

*From:* Moher D, Liberati A, Tetzlaff J, Altman DG, The PRISMA Group (2009). Preferred Reporting Items for Systematic Reviews and Meta-Analyses: The PRISMA Statement. PLoS Med 6 (6): e1000097. doi: 10.1371/journal.pmed1000097

Appendix S3 - PRESS Peer Review of Electronic Search Strategies

| Searcher: Fabiana Lisboa | Email: fabianachristinalisbon@gmail.com |
| --- | --- |
| Date submitted: 27/06/2021 | Date requested by: 02.07.2021  *[Maximum = 5 working days]* |

PEER REVIEW ASSESSMENT: THIS SECTION TO BE FILLED IN BY THE REVIEWER

| Reviewer: Ana Claudia Morais Godoy Figueiredo | Email:[**aninha_m_godoy@hotmail.com**](mailto:aninha_m_godoy@hotmail.com) | | Date completed:  **02.07.2021** |
| --- | --- | --- | --- |
| 1. TRANSLATION | | | |
|  | A. No revisions | **x x** |  |
|  | B. Suggested revision (s) | **□** |  |
|  | C. Revision (s) required | **□** |  |

If “B” or “C,” please provide an explanation or example:

|  |
| --- |

| **2. BOOLEAN AND PROXIMITY OPERATORS** | | | |
| --- | --- | --- | --- |
|  | A. No revisions | **x x** |  |
|  | B. Suggested revision (s) | **□** |  |
|  | C. Revision (s) required | **□** |  |

If “B” or “C,” please provide an explanation or example:

|  |
| --- |

| **3. SUBJECT HEADINGS** | | | |
| --- | --- | --- | --- |
|  | A. No revisions | **x x** |  |
|  | B. Suggested revision (s) | **□** |  |
|  | C. Revision (s) required | **□** |  |

If “B” or “C,” please provide an explanation or example:

|  |
| --- |

| **4. TEXT WORD SEARCHING** | | | |
| --- | --- | --- | --- |
|  | A. No revisions | **□** |  |
|  | B. Suggested revision (s) | **□** |  |
|  | C. Revision (s) required | **□** |  |

If “B” or “C,” please provide an explanation or example:

|  |
| --- |

| **5. SPELLING, SYNTAX, AND LINE NUMBERS** | | | |
| --- | --- | --- | --- |
|  | A. No revisions | **□** |  |
|  | B. Suggested revision (s) | **x x** |  |
|  | C. Revision (s) required | **□** |  |

If “B” or “C,” please provide an explanation or example:

| I suggest that you review the descriptors according to indexed and similar terms. It is important that all similar terms are included in the search strategy. |
| --- |

| **6. LIMITS AND FILTERS** | | | |
| --- | --- | --- | --- |
|  | A. No revisions | **□** |  |
|  | B. Suggested revision (s) | **x x** |  |
|  | C. Revision (s) required | **□** |  |

If “B” or “C,” please provide an explanation or example:

| I recommend that you reconstruct the search strategy by presenting the filters for the mesh term (according to the indexed term) and the title and abstract to make the search more specific. Do not leave the terms free, this makes the search very broad and with a large number of articles that will be of little use for analysis. |
| --- |

| **7. OVERALL EVALUATION (Note: If one or more “revision required” is noted above, the**  **response response must be “revisions required”.)** | | | |
| --- | --- | --- | --- |
|  | A. No revisions | **□** |  |
|  | B. Suggested revision (s) | **x x** |  |
|  | C. Revision (s) required | **□** |  |

Additional comments:

| Review the PICO structure, applying a search line for each item as needed. |
| --- |

Appendix S4 - Search strategy for each database

| **Database** | **Search Strategy** |
| --- | --- |
| PUBMED | **(((((((((((((((((((((((((((((((((Breast Neoplasm [Title/Abstract]) OR (Breast Neoplasm [MeSH Terms])) OR (Breast Cancer [Title/Abstract])) OR (Breast Cancer [MeSH Terms])) OR (Neoplasm, Breast [Title/Abstract])) OR (Breast Tumors [Title/Abstract ]])) OR (Breast Tumor [Title/Abstract])) OR (Tumor, Breast [Title/Abstract])) OR (Tumors, Breast [Title/Abstract])) OR (Neoplasms, Breast [Title/Abstract])) OR (Cancer, Breast [Title/Abstract])) OR (Mammary Cancer [Title/Abstract])) OR (Cancer, Mammary [Title/Abstract])) OR (Cancer, Mammary [Title/Abstract])) OR (Mammary Cancers [Title/Abstract])) OR (Malignant Neoplasm of Breast [Title/Abstract])) OR (Breast Malignant Neoplasm [Title/Abstract])) OR (Breast Malignant Neoplasms [Title/Abstract])) OR (Malignant Tumor of Breast [Title/Abstract])) OR (Breast Malignant Tumor [Title/Abstract])) OR (Breast Malignant Tumors [Title/Abstract])) OR (Cancer of Breast [Title/Abstract])) OR (Cancer of the Breast [Title/Abstract])) OR (Mammary Carcinoma, Human [Title/Abstract ]])) OR (Carcinoma, Human Mammary [Title/Abstract])) OR (Carcinomas, Human Mammary [Title/Abstract])) OR (Human Mammary Carcinomas [Title/Abstract])) OR (Mammary Carcinomas, Human [Title/Abstract])) OR (Human Mammary Carcinoma [Title/Abstract])) OR (Mammary Neoplasms, Human [Title/Abstract])) OR (Human Mammary Neoplasm [Title/Abstract])) OR (Human Mammary Neoplasms [Title/Abstract ]])) OR (Neoplasm, Human Mammary [Title/Abstract])) OR (Neoplasms, Human Mammary [Title/Abstract])) OR (Mammary Neoplasm, Human [Title/Abstract])) OR (Breast Carcinoma [Title/Abstract ]])) OR (Breast Carcinomas [Title/Abstract])) OR (Carcinoma, Breast [Title/Abstract])) OR (Carcinomas, Breast [Title/Abstract])) AND (((((((((((Mammaplasty [MeSH Terms]) OR (Mammaplasty [Title/Abstract])) OR (Mammaplasties [Title/Abstract])) OR (Mammoplasty [Title/Abstract])) OR (Mammoplasties [Title/Abstract])) OR (Breast Reconstruction [Title/Abstract])) OR (Breast Reconstructions [Title/Abstract])) OR (Reconstruction, Breast [Title/Abstract])) OR (Rec onstructions, Breast [Title/Abstract])) OR (Oncoplastic Surgery [Title/Abstract])) OR (Oncoplastic [Title/Abstract])) OR (mammaplast*[Title/Abstract])) OR (mammoplast*[Title/Abstract ]))) OR (((((((((((((((((((((((((((((((Mastectomies, Segmental [Title/Abstract]) OR (Mastectomies, Segmental [MeSH Terms])) OR (Segmental Mastectomies [Title/Abstract])) OR (Segmental Mastectomy [Title/Abstract])) OR (Local Excision Mastectomy [Title/Abstract])) OR (Local Excision Mastectomies [Title/Abstract])) OR (Mastectomies, Local Excision [Title/Abstract])) OR (Mastectomy, Local Excision [Title/Abstract])) OR (Segmentectomy [Title/Abstract])) OR (Segmentectomies [ Title/Abstract])) OR (Partial Mastectomy [Title/Abstract])) OR (Mastectomies, Partial [Title/Abstract])) OR (Mastectomy, Partial [Title/Abstract])) OR (Partial Mastectomies [Title/Abstract]))) OR (Limited Resection Mastectomy [Title/Abstract])) OR (Limited Resection Mastectomies [Title/Abstract])) OR (Mastectomies, Limited Resection [Title/Abstract])) OR (Mastectomy, Limited Resection [Title/Abstract])) OR (Lumpectomy [Title/Abstract])) OR (Lumpectomies [Title/Abstract])) OR (Breast-Conserving Surgery [Title/Abstract])) OR (Breast Conserving Surgery [Title/Abstract])) OR (Breast Quadrantectomy [Title/Abstract])) OR (Breast Quadrantectomies [Title/Abstract])) OR (Quadrantectomies, Breast [Title/Abstract])) OR (Quadrantectomy, Breast [Title/Abstract] ]])) OR (Surgery, Breast-Conserving [Title/Abstract])) OR (Breast-Conserving Surgeries [Title/Abstract])) OR (Surgeries, Breast-Conserving [Title/Abstract])) OR (Surgery, Breast Conserving [Title/Abstract])) OR (Breast Conservation Therapy [Title/Abstract])) OR (Breast Conservation Therapies [Title/Abstract])) OR (Conservation Therapies, Breast [Title/Abstract])) OR (Conservation Therapy, Breast [Title/Abstract])) OR (Breast-Sparing Surgery [Title/Abstract])) OR (Breast Sparing Surgery [Title/Abstract])) OR (Breast-Sparing Surgeries [Title/Abstract])) OR (Surgeries, Breast -Sparing [Title/Abstract])) OR (Surgery, Br east-Sparing [Title/Abstract]))) AND (((Patient Satisfaction [Title/Abstract]) OR (Patient Satisfaction [MeSH Terms])) OR (Satisfaction, Patient [Title/Abstract]))** |
| EMBASE | ('breast reconstruction'/exp OR 'breast reconstruction' OR (('breast'/exp OR breast) AND ('reconstruction'/exp OR reconstruction)) OR 'plastic surgery'/exp OR 'plastic surgery' OR ((' plastic '/exp OR plastic) AND (' surgery '/exp OR surgery)) OR' oncoplastic surgery '/exp OR' oncoplastic surgery 'OR (oncoplastic AND (' surgery '/exp OR surgery)) OR' therapeutic mammoplasty 'OR (therapeutic AND ('mammoplasty'/exp OR mammoplasty)) OR 'therapeutic mammaplasty' OR (therapeutic AND ('mammaplasty'/exp OR mammaplasty)) OR 'oncoplastic breast surgery'/exp OR 'oncoplastic breast surgery' OR (oncoplastic AND ('breast'/exp OR breast) AND ('surgery'/exp OR surgery)) OR 'oncoplastic breast conserving surgery' OR (oncoplastic AND ('breast'/exp OR breast) AND conserving AND ('surgery'/exp OR surgery)) OR oncoplastic) AND ('breast cancer'/exp OR 'breast cancer' OR (('breast'/exp OR breast) AND ('cancer'/exp OR cancer)) OR 'breast neoplasm' OR ((' breast/exp OR breast) AND ('neoplasm'/exp OR neoplasm)) OR 'b reast tumor '/exp OR' breast tumor 'OR ((' breast '/exp OR breast) AND (' tumor '/exp OR tumor))) |
| Clinical Trials | Breast reconstruction, oncoplastic, patient satisfaction, breast cancer |
| Web of science | (((Breast Neoplasms) OR (mh: (Breast Neoplasms)) OR (Breast Neoplasm) OR (Neoplasm, Breast) OR (Breast Tumors) OR (Breast Tumor) OR (Tumor, Breast) OR (Tumors, Breast) OR (Neoplasms, Breast) OR (Breast Cancer) OR (Cancer, Breast) OR (Mammary Cancer) OR (Cancer, Mammary) OR (Cancer, Mammary) OR (Mammary Cancer) OR (Malignant Neoplasm of Breast) OR (Breast Malignant Neoplasm) OR (Breast Malignant Neoplasms) OR (Malignant Tumor of Breast) OR (Breast Malignant Tumor) OR (Breast Malignant Tumors) OR (Cancer of Breast) OR (Cancer of the Breast) OR (Mammary Carcinoma, Human) OR (Carcinoma, Human Mammary) OR (Carcinomas, Human Mammary) OR (Human Mammary Carcinomas) OR (Mammary Carcinomas, Human) OR (Human Mammary Carcinoma) OR (Mammary Neoplasms, Human) OR (Human Mammary Neoplasm) OR (Human Mammary Neoplasms) OR (Neoplasm, Human Mammary) OR (Neoplasms, Human Mammary) OR (Mammary Neoplasm, Human) OR (Breast Carcinoma) OR (Breast Carcinomas) OR (Carcinoma, Breast) OR (Carcinomas, Breast))) AND ((((Mammaplasty) OR (mh: (Mammaplasty)) OR (Mammaplasties) OR (Mammoplasty) OR (Mammoplasties) OR (Breast Reconstruction) OR (Breast Reconstructions) OR (Reconstruction, Breast) OR (Reconstructions, Breast) OR (Oncoplastic Surgery) OR (Oncoplastic) OR (mammaplast*))) OR (((Mastectomy, Segmental) OR (mh: (Mastectomy, Segmental)) OR (Mastectomies, Segmental) OR (Segmental Mastectomies) OR (Segmental Mastectomy) OR (Local Excision Mastectomy) OR (Local Excision Mastectomies) OR (Mastectomies, Local Excision) OR (Mastectomy, Local Excision) OR (Segmentectomy) OR (Segmentectomies) OR (Partial Mastectomy) OR (Mastectomies, Partial) OR (Mastectomy, Partial) OR (Partial Mastectomies) OR (Limited Resection Mastectomy) OR (Limited Resection Mastectomies) OR (Mastectomies, Limited Resection) OR (Mastectomy, Limited Resection) OR (Lumpectomy) OR (Lumpectomies) OR (Breast-Conserving Surgery) OR (Breast Conserving Surgery) OR (Breast Quadrantectomy) OR (Breast Quadrantectomies) OR (Quadrantectomies, Br east) OR (Quadrantectomy, Breast) OR (Surgery, Breast-Conserving) OR (Breast-Conserving Surgeries) OR (Surgeries, Breast-Conserving) OR (Surgery, Breast Conserving) OR (Breast Conservation Therapy) OR (Breast Conservation Therapies) OR (Conservation Therapies, Breast) OR (Conservation Therapy, Breast) OR (Breast-Sparing Surgery) OR (Breast Sparing Surgery) OR (Breast-Sparing Surgeries) OR (Surgeries, Breast-Sparing) OR (Surgery, Breast-Sparing))) AND (((Patient Satisfaction) OR (mh: (Patient Satisfaction)) OR (Satisfaction, Patient))) |
| Scopus | (((TITLE-ABS-KEY (breast AND neoplasm) OR TITLE-ABS-KEY (neoplasm, AND breast) OR TITLE-ABS-KEY (breast AND tumors) OR TITLE-ABS-KEY (breast AND tumor) OR TITLE-ABS -KEY (tumor, AND breast) OR TITLE-ABS-KEY (tumors, AND breast) OR TITLE-ABS-KEY (neoplasms, AND breast) OR TITLE-ABS-KEY (breast AND cancer) OR TITLE-ABS-KEY (cancer, AND breast) OR TITLE-ABS-KEY (mammary AND cancer) OR TITLE-ABS-KEY (cancer, AND mammary) OR TITLE-ABS-KEY (cancers, AND mammary) OR TITLE-ABS-KEY (mammary AND cancers) OR TITLE-ABS-KEY (malignant AND neoplasm AND of AND breast) OR TITLE-ABS-KEY (breast AND malignant AND neoplasm) OR TITLE-ABS-KEY (breast AND malignant AND neoplasms) OR TITLE-ABS-KEY (malignant AND AND tumor AND OF AND breast) OR TITLE-ABS-KEY (breast AND malignant AND tumor) OR TITLE-ABS-KEY (breast AND malignant AND tumors) OR TITLE-ABS-KEY (cancer AND of AND brea st) OR TITLE-ABS-KEY (cancer AND of AND the breast) OR TITLE-ABS-KEY (mammary AND carcinoma, AND human) OR TITLE-ABS-KEY (carcinoma, AND human AND mammary) OR TITLE-ABS- KEY (carcinomas, AND human AND mammary) OR TITLE-ABS-KEY (human AND mammary AND carcinomas) OR TITLE-ABS-KEY (mammary AND carcinomas, AND human) OR TITLE-ABS-KEY (human AND mammary AND carcinoma) OR TITLE-ABS-KEY (mammary AND neoplasms, AND human) OR TITLE-ABS-KEY (human AND mammary AND neoplasm) OR TITLE-ABS-KEY (human AND mammary AND neoplasms) OR TITLE-ABS-KEY (neoplasm, AND human AND AND mammary) OR TITLE-ABS-KEY (neoplasms, AND human AND mammary) OR TITLE-ABS-KEY (mammary AND neoplasm, AND human) OR TITLE-ABS-KEY (breast AND carcinoma) OR TITLE-ABS-KEY (breast AND carcinomas) OR TITLE-ABS-KEY (carcinoma, AND breast) OR TITLE-ABS-KEY (carcinomas, AND breast))) AND ((TITLE-ABS-KEY (mammaplasty) OR TITLE-ABS-KEY (mammaplasties) OR TITLE-ABS-KEY (mammoplasty) OR TITLE-ABS-KEY (mammoplasties) OR TITLE-ABS-KEY (breast AND reconstruction) OR TITLE-ABS-KEY (breast AND reconstructions) OR TITLE-ABS-KEY (reconstruction, AND breast) OR TITLE-ABS-KEY (reconstructions, AND breast) OR TITLE-ABS-KEY (oncoplastic AND surgery) OR TITLE-ABS-KEY (oncoplastic))) OR ((TITLE-ABS-KEY (mastectomy, AND segmental) OR TITLE-ABS-KEY (mastectomy, AND segmental) OR TITLE-ABS-KEY (segmental AND mastectomies) OR TITLE-ABS-KEY (segmental AND mastectomy) OR TITLE-ABS- KEY (local AND excision AND mastectomy) OR TITLE-ABS-KEY (local AND excision AND mastectomies) OR TITLE-ABS-KEY (mastectomies, AND local AND excision) OR TITLE-ABS-KEY (mastectomy, AND local AND excision) OR TITLE-ABS-KEY (segmentectomy) OR TITLE-ABS-KEY (segmentectomy) OR TITLE-ABS-KEY (partial AND mastectomy) y) OR TITLE-ABS-KEY (mastectomies, AND partial) OR TITLE-ABS-KEY (mastectomy, AND partial) OR TITLE-ABS-KEY (partial AND mastectomies) OR TITLE-ABS-KEY (limited AND resection AND mastectomy) OR TITLE-ABS-KEY (limited AND resection AND mastectomies) OR TITLE-ABS-KEY (mastectomies, AND limited AND resection) OR TITLE-ABS-KEY (mastectomy, AND limited AND resection) OR TITLE-ABS-KEY (lumpectomy) OR TITLE-ABS-KEY (lumpectomies) OR TITLE-ABS-KEY (breast-conserving AND surgery) OR TITLE-ABS-KEY (breast AND conserving AND surgery) OR TITLE-ABS-KEY (breast AND quadrantectomy) OR TITLE-ABS -KEY (breast AND quadrantectomies) OR TITLE-ABS-KEY (quadrantectomies, AND breast) OR TITLE-ABS-KEY (quadrantectomy, AND breast) OR TITLE-ABS-KEY (surgery, AND breast-conserving) OR TITLE-ABS- KEY (breast-conserving AND surgeries) OR TITLE-ABS-KEY (surgeries, AND breast-conserving) OR TITLE-ABS-KE Y (surgery, AND breast AND conserving) OR TITLE-ABS-KEY (breast AND conservation AND therapy) OR TITLE-ABS-KEY (breast AND conservation AND therapies) OR TITLE-ABS-KEY (conservation AND therapies, AND breast) OR TITLE-ABS-KEY (conservation AND therapy, AND breast) OR TITLE-ABS-KEY (breast-sparing AND surgery) OR TITLE-ABS-KEY (breast AND sparing AND surgery) OR TITLE-ABS-KEY (breast-sparing AND surgeries) OR TITLE-ABS-KEY (surgeries, AND breast-sparing) OR TITLE-ABS-KEY (surgery, AND breast-sparing))) AND ((TITLE-ABS-KEY (patient AND satisfaction) OR TITLE-ABS- KEY (satisfaction, AND patient))) |
| VHL  Oppen Gray | (((Breast Neoplasms) OR (mh: (Breast Neoplasms)) OR (Breast Neoplasm) OR (Neoplasm, Breast) OR (Breast Tumors) OR (Breast Tumor) OR (Tumor, Breast) OR (Tumors, Breast) OR (Neoplasms, Breast) OR (Breast Cancer) OR (Cancer, Breast) OR (Mammary Cancer) OR (Cancer, Mammary) OR (Cancer, Mammary) OR (Mammary Cancer) OR (Malignant Neoplasm of Breast) OR (Breast Malignant Neoplasm) OR (Breast Malignant Neoplasms) OR (Malignant Tumor of Breast) OR (Breast Malignant Tumor) OR (Breast Malignant Tumors) OR (Cancer of Breast) OR (Cancer of the Breast) OR (Mammary Carcinoma, Human) OR (Carcinoma, Human Mammary) OR (Carcinomas, Human Mammary) OR (Human Mammary Carcinomas) OR (Mammary Carcinomas, Human) OR (Human Mammary Carcinoma) OR (Mammary Neoplasms, Human) OR (Human Mammary Neoplasm) OR (Human Mammary Neoplasms) OR (Neoplasm, Human Mammary) OR (Neoplasms, Human Mammary) OR (Mammary Neoplasm, Human) OR (Breast Carcinoma) OR (Breast Carcinomas) OR (Carcinoma, Breast) OR (Carcinomas, Breast))) AND ((((Mammaplasty) OR (mh: (Mammaplasty)) OR (Mammaplasties) OR (Mammoplasty) OR (Mammoplasties) OR (Breast Reconstruction) OR (Breast Reconstructions) OR (Reconstruction, Breast) OR (Reconstructions, Breast) OR (Oncoplastic Surgery) OR (Oncoplastic) OR (mammaplast*))) OR (((Mastectomy, Segmental) OR (mh: (Mastectomy, Segmental)) OR (Mastectomies, Segmental) OR (Segmental Mastectomies) OR (Segmental Mastectomy) OR (Local Excision Mastectomy) OR (Local Excision Mastectomies) OR (Mastectomies, Local Excision) OR (Mastectomy, Local Excision) OR (Segmentectomy) OR (Segmentectomies) OR (Partial Mastectomy) OR (Mastectomies, Partial) OR (Mastectomy, Partial) OR (Partial Mastectomies) OR (Limited Resection Mastectomy) OR (Limited Resection Mastectomies) OR (Mastectomies, Limited Resection) OR (Mastectomy, Limited Resection) OR (Lumpectomy) OR (Lumpectomies) OR (Breast-Conserving Surgery) OR (Breast Conserving Surgery) OR (Breast Quadrantectomy) OR (Breast Quadrantectomies) OR (Quadrantectomies, Br east) OR (Quadrantectomy, Breast) OR (Surgery, Breast-Conserving) OR (Breast-Conserving Surgeries) OR (Surgeries, Breast-Conserving) OR (Surgery, Breast Conserving) OR (Breast Conservation Therapy) OR (Breast Conservation Therapies) OR (Conservation Therapies, Breast) OR (Conservation Therapy, Breast) OR (Breast-Sparing Surgery) OR (Breast Sparing Surgery) OR (Breast-Sparing Surgeries) OR (Surgeries, Breast-Sparing) OR (Surgery, Breast-Sparing))) AND (((Patient Satisfaction) OR (mh: (Patient Satisfaction)) OR (Satisfaction, Patient))) (Breast Neoplasms OR Breast Neoplasm OR Neoplasm, Breast OR Breast Tumors OR Breast Tumor OR Tumor, Breast OR Tumors, Breast OR Neoplasms, Breast OR Breast Cancer OR Cancer, Breast OR Mammary Cancer OR Cancer, Mammary OR Cancer, Mammary OR Mammary Cancer OR Malignant Neoplasm of Breast OR Breast Malignant Neoplasm OR Breast Malignant Neoplasms OR Malignant Tumor of Breast OR Breast Malignant Tumor OR Breast Malignant Tumors OR Cancer of Breast OR Cancer of the Breast OR Mammary Carcinoma, Human OR Carcinoma, Human Mammary OR Carcinomas, Human Mammary OR Human Mammary Carcinomas OR Mammary Carcinomas, Human OR Human Mammary Carcinoma OR Mammary Neoplasms, Human OR Human Mammary Neoplasm OR Human Mammary Neoplasms OR Neoplasm, Human Mammary OR Neoplasms, Human Mammary OR Mammary Neoplasm, Human OR Breast Carcinoma OR Breast Carcinomas OR Carcinoma, Breast OR Carcinomas, Breast) AND (Mammaplasty OR Mammaplasties OR Mammoplasty OR Mammoplasties OR Breast Reconstruction OR Breast Reconstructions OR Reconstruction, Breast OR Reconstructions, Breast OR Oncoplastic Suergery OR Oncoplastic) OR (Mastectomy, Segmental OR Mastectomies, Segmental OR Segmental Mastectomies OR OR Segmental Mastectomy OR OR Local Excision Mastectomy OR Local Excision Mastectomies OR Mastectomies, Local Excision OR Mastectomy, Local Excision OR Segmentectomy OR OR Segmentectomies OR OR Partial Mastectomy OR OR Mastectomies, Partial OR Mastectomy, Partial OR OR Partial Mastectomies OR OR Limited Resection Mastectomy OR Limited Resection Mastectomies OR Mastectomies, Limited Resection OR Mastectomy, Limited Resection OR Lumpectomy OR Lumpectomies OR Breast-Conserving Surgery OR OR Breast Conserving Surgery OR Breast Quadrantectomy OR OR Breast Quadrantectomies OR Quadrantectomies, Breast OR Quadrantectomy, Breast OR Surgery, Breast-Conserving OR OR Breast-Conserving Surgeries OR Surgeries, Breast-Conserving OR Surgery, Breast Conservation OR OR Breast Conservation Therapy OR Breast Conservation Therapies OR Conservation Therapies, Breast OR OR Conservation Therapy, Breast OR Breast-Sparing Surgery OR OR Breast Sparing Surgery OR OR Breast-Sparing Surgeries OR Surgeries, Breast-Sparing OR Surgery, Breast-Sparing) AND (Patient Satisfaction OR Satisfaction, Patient) |
|  |  |
|  |  |
